# Supplementary material for: Site Mutation Improves the Expression and Antimicrobial Properties of Fungal Defense
Source: Antibiotics (Basel). 2023 Aug 3;12(8):1283. doi: 10.3390/antibiotics12081283 (PMC10451632; doi:10.3390/antibiotics12081283)
Supplement: Supplementary file 1 [file antibiotics-12-01283-s001.zip › antibiotics-2482718-supplementary.pdf]

Site mutation and screening of fungal defense with high expression and bioactivity

Ya Hao [1, 2, 3*], Da Teng [1, 2, 3], Ruoyu Mao [1, 2, 3], Na Yang [1, 2, 3], and Jianhua Wang [1, 2, 3*]

1, Gene Engineering Laboratory, Feed Research Institute, Chinese Academy of Agricultural Sciences, Beijing 100081, China

2, Innovative Team of Antimicrobial Peptides and Alternatives to Antibiotics, Feed Research Institute, Chinese Academy of Agricultural Sciences, Beijing 100081, China

3, Key Laboratory of Feed Biotechnology, Ministry of Agriculture and Rural Affairs, Beijing 100081, China

* Corresponding authors

(1)Ya Hao, email address: haoya@caas.cn

(2)Jianhua Wang, as the sole contact during this submission

Feed Research Institute, Chinese Academy of Agricultural Sciences,

12 Zhongguancun Nandajie St., Haidian District, Beijing 100081, China

E-mail address: wangjianhua@caas.cn

Phone/Fax: +86-10-82106081, +86-10-82106079

A

Parameters	Glucose	Ca^{2+}	Phosphorous	Urea Nitrogen	Creatinine	Total protein	Albumin	Globulin	ALB/GLO	AST	ALT	AST/ALT
Unit	mmol/L	mmol/L	mmol/L	mmol/L	μmol/L	g/L	g/L	g/L		IU/L	IU/L	
CK	1.55±0.09	1.39±0.02	3.03±0.11	9.77±0.58	94.82±2.91	51.32±1.74	31.84±1.41	19.48±0.86	1.64±0.09	27.43±1.12	52.12±1.54	0.53±0.01
PN7	1.61±0.05	1.36±0.02	3.08±0.10	10.13±0.59	97.08±6.55	48.99±1.60	30.14±0.66	18.86±1.15	1.61±0.09	32.85±1.56	60.06±5.25	0.56±0.03

B

Parameters	RBC	HBG	HCT	MCV	MCH	MCHC	PLT	MPV	PDW	PCT	RDW-CV	RDW-SD
Unit	10^{12}/L	g/L	%	fL	pg	g/L	10^{9}/L	fL	%	%	10^{9}/L	%
CK	9.23±0.27	120.00±2.35	38.85±1.20	40.13±1.21	12.88±0.26	32.53±0.69	847.00±26.62	6.48±0.23	15.73±0.29	0.06±0.01	17.85±0.15	42.35±2.42
PN7	8.84±0.31	115.25±4.85	38.60±1.42	40.27±0.38	12.73±0.42	31.95±0.33	798.25±40.12	6.58±0.31	16.13±0.40	0.03	17.58±0.39	40.93±1.20

C

Parameters	WBC	NEUT	LYM	MONO	EO	BASO	NEUT%	LYM%	MONO%	EO%	BASO%
Unit	10^{9}/L	10^{9}/L	10^{9}/L	10^{9}/L	10^{9}/L	10^{9}/L	%	%	%	%	%
CK	11.39±0.73	2.86±0.31	7.40±0.25	0.57±0.13	0.28±0.03	0.29±0.03	24.88±1.26	65.37±224	4.83±0.96	2.42±0.25	2.50±0.14
PN7	13.20±2.01	3.66±0.97	8.16±0.66	0.76±0.24	0.35±0.08	0.27±0.07	26.26±3.30	63.98±4.79	5.25±1.11	2.58±0.25	1.94±0.25

Figure S1 Serum biochemical indices (A) and whole-blood cell profiles (B and C) of mice (ICR mice (6-8 weeks old, male, n = 5 per group; Charles River, Beijing) were intra-peritoneally injected with PN7 (10 mg/kg, body weight) for 6 days). Data are representative of three biological replicates and the mean was shown.
